# Monitoring of coagulation in patients after abdominal cancer surgery

**DOI:** 10.1186/cc9855

**Published:** 2011-03-11

**Authors:** O Tarabrin, V Suslov, S Kalinchuk, S Shcherbakov, D Gavrychenko

**Affiliations:** 1 Odessa National Medical University, Odessa, Ukraine; 2 Odessa Regional Clinical Hospital, Odessa, Ukraine

## Introduction

Despite the evidence of perioperative hypercoagulability in cancer patients, there are no consistent data evaluating the extent, duration, and specific contribution of platelets and procoagulatory proteins by *in vitro *testing. This study compared efficacy of haemo-viscoelastography (HVG) versus thromboelastography for monitoring of coagulation imbalance.

## Methods

A total of 536 patients undergoing open surgery for abdominal cancer received HVG, a viscoelastic test, which measures clot formation and includes information on the cellular as well as the plasmatic coagulation system. We examined the efficacy of a variety of coagulation tests. A complete coagulation screen activated clotting time (ACT), thromboelastography (TEG) and HVG were performed before surgery, at the end of surgery, and bemiparin anticoagulation monitoring on postoperative days 1, 2, 3, and 7. These were analyzed for the reaction time and the maximal amplitude (MA).

## Results

We calculated the elastic shear modulus of standard MA and HVG MA, which reflect the total clot strength and procoagulatory protein component, respectively. The difference was an estimate of the platelet component. There was a l6% perioperative increase of standard MA, corresponding to a 49% increase of HVG MA (*P *< 0.05) and a 79 to 85% contribution of the calculated platelet component to HVG MA. We conclude that serial standard thromboelastography and HVG may reveal the independent contribution of platelets and procoagulatory proteins to clot strength. Using multiple linear regressions, all coagulation, TEG and HVG variabilities were used to model postoperative hypercoagulation. Results showed that some components of the TEG failed to identify hypercoagulation (*r *< 0.2, *P *> 0.75). However, three components of the routine coagulation assay, including the bleeding time, prothrombin time, and platelet count, could be modeled to show prolonged postoperative hyper-coagulability (*P *< 0.01). We conclude that all components of the HVG reflect postoperative coagulopathies; these results suggest that it may be useful in determining the coagulation status of cancer patients perioperatively.

## Conclusions

Postoperative hypercoagulability, occurring for at least 1 week after major cancer abdominal surgery, may be demonstrated by HVG. Hypercoagulability is not reflected completely by standard coagulation monitoring and TEG and seems to be predominantly caused by increased platelet reactivity.

